# Guillain-Barré Syndrome due to CMV Reactivation after Cardiac Transplantation

**DOI:** 10.1155/2012/506290

**Published:** 2012-02-14

**Authors:** Christina Maria Steger, Herwig Antretter, Daniel Höfer

**Affiliations:** ^1^Department of Pathology, Innsbruck Medical University, Müllerstraße 44, 6020 Innsbruck, Austria; ^2^Department of Cardiac Surgery, Innsbruck Medical University, Anichstrasse 35, 6020 Innsbruck, Austria

## Abstract

A 40-year-old male patient suffered from end-stage heart failure due to ischemic cardiomyopathy and received orthotopic cardiac transplantation in June 2005. The instantaneous postoperative course was uneventful, but, seven months later, he suffered from paralysis in the lower extremities finally resulting in quadriplegia and was admitted to hospital. After laboratory testings the diagnosis of a Guillain-Barré syndrome due to cytomegalovirus reactivation was confirmed.

## 1. Introduction

Cytomegalovirus (CMV) infection is one of the most important infectious complications of solid-organ transplantation [1] and is responsible for serious, life-threatening diseases in patients infected with human immunodeficiency virus (HIV) [2]. CMV infection, defined as a significant rise in the titre of CMV-specific antibodies, occurs in 44–85% of kidney, heart, and liver transplant recipients, commonly within the first 3 months after transplantation, when immunosuppression is most intense [3, 4]. Cytomegalovirus is a member of the b-herpes-virus group and is characterised by its strict species specificity, long life cycle, and lifetime persistence within the host [5].

Guillain-Barré syndrome (GBS) is a rare acute inflammatory demyelinating polyneuropathy (AIDP) with an incidence of 1 or 2 people per 100,000, an autoimmune disorder affecting the peripheral nervous system, usually triggered by an acute infectious process and exhibiting as an ascending paralysis noted by weakness in the legs that spreads to the upper limbs and the face along with complete loss of deep tendon reflexes. The syndrome was named after the French physicians Guillain, Barré, and Strohl, who were the first to describe it in 1916. It can occur at any age but is most common between ages 30 and 50 and affects both sexes equally. GBS follows viral infection in more than 50% of cases. The most common antecedent infection is the bacteria *Campylobacter jejuni* (4–66%), followed by *Cytomegalovirus* (5–15%), Epstein-Barr virus (2–10%), Mycoplasma pneumonia (1–5%), Varicella-zoster virus, and acute HIV infection. GBS is also seen in a higher-than-expected rate in patients with sarcoidosis, systemic lupus erythematosus, lymphoma, HIV infection, Lyme disease, and solid tumors, and it may be triggered by mononucleosis, Hodgkin's disease, and rarely rabies or influenza immunizations.

GBS results from an aberrant immune response that somehow mistakenly attacks the nerve tissue of its host, most probably by recognizing a molecular similar epitope mechanism. Circulating antiganglioside antibodies (e.g., GM1, GM2, and GQ1B) found in particular subtypes suggest a molecular mimicry mechanism stimulated by infection. Immune reactions against these epitopes result in acute inflammatory demyelinating neuropathy or acute axonal forms. However, 60% of cases do not have a known cause; one study suggests that some cases are triggered by the influenza virus or by an immune reaction to the influenza virus.

## 2. Case Presentation

In June 2005, a 40-year-old male patient received orthotopic heart transplantation for end-stage cardiac disease secondary to ischemic cardiomyopathy. His past medical history included a myocardial infarction in December 2002 and the implantation of a cardioverter defibrillator in 2003. Preoperative echocardiography showed a grade 3 mitral valve insufficiency, a grade 2 tricuspidal valve insufficiency (NYHA III), and an akinetic anterior ventricular wall with an anterior wall aneurysm. The left ventricular function was highly restricted with an ejection fraction of 30%.

The immediate postoperative course was uneventful. The initial induction protocol after cardiac transplantation consisted of antithymocyte globulin (ATG) for 5 days (total dose 500 mg), aprednisolone in decreasing dosage, and imurek according to the level of leucocytes. Since day 4 after transplantation cyclosporine A treatment was introduced gradually. For CMV mismatch (CMV-positive recipient and CMV-negative donor) the patient was preemptively treated with Cymevene for 14 days after transplantation. Additionally he received valganciclovir per os since day 15 following transplantation. Postoperatively obtained endomyocardial biopsies were all graded 0R (ISHLT 2004), except one biopsy 3 weeks after transplantation (grade IR).

In January 2006, 7 months after transplantation, he developed an unusual lower-extremity weakness and tingling, finally resulting in quadriplegia and decreased vital capacity requiring mechanical ventilation. Electromyography (EMG) and spinal tap confirmed the diagnosis of GBS (axonal form). CMV early antigen pp65 was positive in polymerase chain reaction and showed 1445 copies CMV/mL, GM1 (IgM, IgG), and GQ1b (IgM, IgG), and Sulfatide IgM antibody levels of the plasma were negative. The patient received 5 cycles of plasmapheresis and Cymevene for 3 weeks. In the following months he achieved nearly full physical recovery.

20 months later, in September 2007, he was admitted to hospital because of pneumonia and received antibiotic treatment. The routinely obtained endomyocardial biopsies revealed a grade IR cellular rejection and the respiratory situation improved, but 10 days after admission he collapsed in the bathroom and died due to sudden cardiac death.

## 3. Discussion

CMV infections still have a substantial impact on graft and patient survival in solid organ transplantation. Three-quarters of all patients undergoing solid organ transplantation are believed to experience new infection or reactivation of latent cytomegalovirus (CMV). Of all patients who develop clinical manifestations of CMV infection more than 90% do so within 1–6 months after transplantation, and 60% of the febrile episodes during this period are due to CMV infections. Most of the patients have the so-called self-limiting syndrome, consisting of fever (often spiking), arthralgia, leukopenia and/or thrombocytopenia, and abnormal liver enzymes [6, 7].

Aside from this self-limiting syndrome, CMV may cause a myriad of symptoms in the grafted patient: gastrointestinal symptoms including gastrointestinal ulcers, pancreatitis, granulomatous hepatitis, pneumatosis intestinalis, lymphadenopathy, hepatosplenomegaly, pericarditis, myocarditis, encephalitis, retinitis, and skin ulcerations associated with vasculitis [6–15].

Risk factors for CMV disease are well recognized and include the following: transplanting a CMV-seronegative patient with a CMV-seropositive donor organ (leading to a primary infection; this is associated with a relative risk of infection >20 times higher than the D-/R-combination). In addition, the recipient's state of immunosuppression plays an important role, determined by the characteristics of the immunosuppressive protocol (type, dose, duration, and timing) as well as various host factors (comorbidity, age, uremia, neutropenia, infections with other immunomodulating viruses, and a high virus load) [6].

As far as the type of immunosuppression is concerned, especially antilymphocyte antibodies and monoclonal antibody preparations such as OKT3 are well known to cause a high incidence of CMV disease when used for either induction or antirejection therapy. And also TNF-a has been suspected to play a role in the reactivation of CMV by stimulation of the CMV major immediate early enhancer/promoter [16, 17].

In contrast to AIDS patients, in whom prolonged CMV viremia is the rule, duration of viremia is usually short in transplanted patients, being mostly confined to the period of maximum immunosuppression, that is, shortly after initiation of antirejection therapy. CMV pneumonia after solid organ transplantation is the manifestation that distinguishes serious illness from the more benign disease, and the condition is associated with high mortality, especially when assisted ventilation is required [6, 7, 18].

GBS has a variety of clinical presentations so that index of suspicion is critical. Typical features are progressive motor weakness and areflexia, often following distal sensory changes. Common presentations include decreased ambulation (or crawling in toddlers), facial weakness, back pain, or sensory changes in the extremities. 85% have a good recovery; ultimate functional recovery depends on the degree of axonal injury, which can be predicted from electrodiagnostic studies in adults. Early prognosticators include the severity of weakness at the disease nadir and fulminant onset. Overall prognosis in children is better than in adults. Complications include respiratory failure, blood pressure dysregulation (hypotension and/or hypertension), urinary retention, aspiration, pain syndromes, deep venous thrombosis, and infection. Death from early respiratory failure, autonomic instability, or other complications occurs in 3–6%.

Despite numerous reports of GBS in recipients of bone marrow transplants, GBS has rarely been reported in recipients of solid organ transplants. Including the present case, only 4 cases of GBS associated with CMV reactivation after cardiac transplantation are published in the medical literature [19, 20] and all of them have much in common (Table 1): all patients were men, all developed GBS within 7 months after transplantation (range 1 week–7 months), all of them received total plasma exchange, and all four patients recovered after illness, although two of them died in the long-term view (one patient died after 13 months because of myocardial infarction, and another one died due to sudden cardiac death 27 months after the diagnosis of GBS).

In conclusion, GBS due to CMV reactivation is a rare neurological complication after cardiac transplantation. With prompt treatment by plasmapheresis or intravenous immunoglobulins and supportive care, the majority of patients suffering from GBS will regain full functional capacity. However, death may occur if severe pulmonary complications and autonomic nervous system problems are present.

## Figures and Tables

**Table 1:** Reported cases of cytomegalovirus-associated Guillain-Barré syndrome after cardiac transplantation [19].

Cases (ref)	Sex/age/race	Transplant Etiology	CMV status Pre-TX D-R	Evidence of active CMV infection	Tx type	Onset post-Tx	Ventilator Support	IVIg	TPE	Maximum grade/final grade

Baldwin [20]	M/61/NS	NS	P-N	Yes	OHT	4 mo	No	Yes	Yes	G4/G1 (died 13 mo, acute MI)
Baldwin [20]	M/62/C	Rheumatic	P-P	Yes	OHT	4.5 mo	No	Yes	Yes	G4/G2
El-Sabrout [19]	M/62/B	Dilated CMP	P-N	Yes	OHT	1 wk	Yes	Yes	Yes	G5/G2
Steger	M/40/C	Ischemic CMP	N-P	Yes	OHT	6 mo	Yes	No	Yes	(died 27 months after Tx, sudden cardiac death)

B: black; C: Caucasian; CMP: cardiomyopathy; D: donor; IVIg: intravenous immunoglobulin; MI: myocardial infarction; mo: months; N: negative; NS: not specified; OHT: orthotopic heart transplantation; P: positive; TPE: total plasma exchange; Tx: transplantation; wk: week.
